# Ptomaines

**Published:** 1888-01

**Authors:** Charles O. Curtman

**Affiliations:** St. Louis, Missouri


### PTOMAINES.

BY CHARLES O. CURTMAN, M. D., ST. LOUIS, MISSOURI.

A report on the present state of our knowledge of ptomaines and their relations to bacteria.

The detection of the poisonous vegetable alkaloids in the dead body has always been considered to be one of the more difficult problems of analytical chemistry. Yet by means of the methods devised by Stas, Otto, Erdmann, Uslar, and Dragendorff, and their various modifications, sufficient of system and accuracy was introduced to regard the results with a general feeling of confidence. If errors and deficiencies occurred now and then, they were placed to the account of individual inaccuracy and want of dexterity rather than that of imperfection in the methods. Some chemical authorities cautioned against wrong conclusions, as, *e. g.*, Sonnenschein against mistakes occasioned by the "well-known substance derived from he liver," but altogether the established methods were relied upon as trustworthy, and expert testimony in criminal cases was fully based upon them. Hence the publication by Selmi, in 1873, of his investigations of cadaveric alkaloids or ptomaines produced amazement and consternation, by destroying at a sudden stroke all confidence in the results of past research in this field. The position of forensic experts became especially painful by creating doubts of the justice of certain decisions in criminal cases.

The discoveries of Selmi were in a short time confirmed by other scientists, and each year increased the number of substances found in dead bodies, which gave with the customary reagents, color changes and precipitates similar to those produced by the well-known alkaloids. There were reactions found closely resembling those of delphinine, curarine, strychnine, etc., and the efforts of analysts were henceforth directed to the discovery of means to distinguish the two classes of substances.

Among the first of them Brouardel and Boutmy proposed the reduction of a solution of ferric chloride and ferricyanide of potassium to the insoluble Prussian blue. They asserted that such reduction was the peculiar property of all ptomaines, while none of the true alkaloids produced it, with the sole exception of morphine. Armand Gautier showed, however, that hyoscyamine, colchicine, nicotine and apomorphine also reduced the ferric ferricyanide solution, though somewhat more slowly than morphine. To this list of reducing vegetable alkaloids C. Tanret added ergotinine, aconitine, digitaline and eserine. In an investigation published several years ago I succeeded in showing that besides morphine and the above mentioned, a number of other alkaloids of opium, as well as brucine, strychnine, cocaine, atropine, cinchonidine and several others reduce the ferric ferricyanide solution with great rapidity. It has since been found that many ptomaines are entirely devoid of such power of reduction, while in many cases this action was due to admixtures of peptone, collidine, etc., to the impure ptomaines as they were generally obtained in sirupy extracts.

In hunting for other means of differentiation, it was found that while most alkaloids are optically active in turning the plane of polarization, ptomaines are altogether inactive. But even this distinction, should it prove to be applicable in every case, avails but little, on account of the minute quantities to which the analyst is so frequently limited.

Still the number of ptomaines kept on increasing, and, based upon coincidences of the reactions with those usually relied upon for alkaloids, many individuals, some poisonous, some innocuous, were described and paraded in the journals. The difficulties of distinguishing them from vegetable alkaloids appeared to multiply to such an extent that forensic chemists began to think they would be driven off of this field of investigation entirely.

Meanwhile an extensive literature on this subject was forming, and the names of T. and H. Husemann, Panum, Selmi, Ciotto, Graebner, Otto, Dragendorff* and others appeared frequently in our professional journals in connection with interesting reports on ptomaines. As a reminder I mention a few items.

In the criminal process against the servant of the Italian general, Gibbone, accused of having poisoned his master, the experts reported that delphinine had been the cause of the general's death. Francesco Selmi saved the defendant from unjust conviction by proving that the reactions erroneously relied upon to prove the presence of delphinine were due to a ptomaine formed by the putrefaction of the dead body.

In the case of the widow Sonzogno, who was believed to have perished by morphine poisoning, Selmi also proved the experts in error, and showed conclusively that a cadaveric base had furnished the reactions which had been mistaken for those of morphine.

In a third case in Verona, strychnine, so easily recognizable by characterstic reactions, was reported as the cause of death, while Selmi proved clearly that strychnine was absent and that some reactions similar to it had been due to a ptomaine.

Shortly before his early death the same distinguished investigator prepared a crystalline substance, resembling curarine from putrefying albumen. The ptomaine which showed such great resemblance to strychnine was also found by Ciotto.

Panum isolated from septicemic material, almost in a pure state, a ptomaine resembling curarine, and ascribed it to the action of bacteria. In animals subjected to its action it produced violent inflammation of the mucous membrane of the small bowel. Bence Jones and Dupré obtained from human and animal livers in an advanced state of decomposition a substance showing blue fluorescence like that of quinine, to which they gave the name of animal chinoidine.

Zuesler and Sonnenschein prepared from putrescent cadavers a substance resembling atropine, which, in experiments on animals, increased the activity of the heart, enlarged the pupil and paralyzed the non-striated muscles of the intestine.

Roersch and Fassbender obtained a ptomaine similar to digitaline; Schwanert, one resembling propylamine. One, approaching in qualities to coniine, was found by Armand Gautier and also by Marquardt and Hager, and was named septicine by the latter. In the criminal case Krebs-Brandes a similar substance was found by Otto accompanying the arsenic that had been administered. In a goose, Brouardel and Boutmy found a ptomaine resembling coniine.

Besides these, many other ptomaines were discovered by various investigators. Not only decomposing animal tissues, but also various parts of plants, furnished ptomaines. Thus Bergmann and Schmiedeberg obtained from putrid yeast a poisonous crystalline substance, which they named sepsine.

A. Poehl, a member of the commission charged by the Russian government with the investigation of the epidemic of ergotism which prevailed so extensively in 1881, found a large amount of ptomaines in flour made from rye infested by the claviceps purpureus.

Many efforts were made to improve the prevailing methods of Otto-Stas and of Dragendorff, so as to insure a more accurate differentiation between alkaloids and ptomaines, but to some extent all failed, and no method yielded the ptomaines in a sufficiently pure, crystallized condition for accurate analysis.

Nencki, of Berne, was the first who obtained from putrid gelatine a chemically pure platinum double salt of a ptomaine. This, on analysis, proved to be collidine, $C_8H_{11}N$, a member of the pyridine series.

Shortly after this, Gautier and Etard prepared parvoline, $C_9H_{13}N$, from putrefying mackerel.

Since 1883, Professor Brieger, of Berlin, has occupied himself considerably with the investigation of the ptomaines, and succeeded in elaborating methods for the isolation of a great number of these interesting substances in a state of absolute purity. He reached his object principally by means of the preparation of the gold and platinum double salts and the picrates of the ptomaines, which crystallize in well-defined forms, and permit absolute purification.

The pure ptomaine salts thus obtained differ materially from the sirupy extracts hitherto supposed to be the pure salts. Many of the alkaloid reactions observed with the impure articles do not occur at all with the pure substances, and were due to admixtures of peptones and other bodies. A number of the compounds, as now studied in their pure state, do not come under the classification of true alkaloids in the present sense, which restricts the name alkaloids to the pyridine derivatives, but prove to be amines, ammonium bases and amido compounds of various composition. Some, in chemical constitution as well as in their toxic effects, resemble *muscarine*, $CHO—CH_2—N(CH_3)_3—OH+H_2O$. This poisonous base occurs together with amanitine (identical with choline from bile) in the fly-agaric, amanita muscaria. It has been artifically prepared by oxidation of the choline, or bilineurine, of hog's bile. These bodies are in close relation with *glycine* (called also glycocoll or amido-acetic acid), among whose derivatives we number *betaine* (called also oxyneurine or lycine), found in beets, the highly poisonous *neurine*, the non-poisonous *neuridine* and many others.

Others of the pure bases discovered by Brieger are di-amines, as, for instance, the *cadaverine*, identical with pentamethylene-diamine, $NH_2—CH_2—CH_2—CH_2—CH_2—CH_2—NH_2$, and *putrescine*, a di-methyl-ethylene-diamine.

In his second report on investigations of the ptomaines, published in 1885, Brieger enumerates the following ptomaines discovered by him in human cadavers:

Choline, $C_5H_{15}NO_2$, a non-poisonous ethylium base.

Neuridine, $C_5H_{14}N_2$, non-poisonous.

Cadaverine, $C_5H_{16}N_2$, a strong reducent.

Putrescine, $C_4H_{12}N_2$.

Saprine, $C_5H_{16}N_2$, strong reducent.

Trimethylamine, $(CH_3)_3$ N.

Mydaleine,————, a strong reducent.

Most of these are diamines of the fatty series.

Besides these bases, other well-known products of putrefaction were found, such as phenol, mostly paired with sulphuric acid, ortho- and para-cressol, indol and scatol, as well as other bodies of the aromatic series.

All of these occur as products of putrefaction, and it appears that within the first few days after death no poisonous base is formed. After the disappearance of the non-poisonous choline, however, compounds are formed of strongly toxic properties, among them especially *neurine*. When a solution of a few milligrammes of this is injected into cats, guinea pigs or rabbits, it rapidly induces fatal poisoning, most especially in the cat. At first copious moisture appears in the nares, then tough mucus in the corners of the mouth, followed by copious salivation, lachrymation, accelerated respiration and dyspnœa. The pulse at first is more frequent, then gradually sinks, and death occurs in diastole, with the heart filled to its utmost capacity. The symptoms are accompanied by increased peristaltic action of the bowels, and contraction of the pupils. Atropine is an effective antidote.

As there was a strong probability that these ptomaines were products of the metamorphosis of protein bodies by the bacteria of putrefaction, several series of experiments were instituted to cultivate them in sterilized protein derivatives, so as to be able to test purified products of various kinds of bacteria. In addition to the basic substances already named, there were found in putrefying fish and horse-flesh, *gadinine*, *ethylene-diamine*, *tri-methyl-amine* and other bases. Recently, Vaughan prepared from milk and cheese the *tyrotoxine*, which, however, thus far has not been obtained of sufficient purity for analysis.

The action of *pathogenic bacteria* was also studied, and ptomaines were obtained, which promised interesting explanation of the symptoms of infectious diseases. After the discovery of pathogenic bacteria and their noxious influence on the living organism of plants and animals, the modus operandi of their deleterious action still remained obscure. It was accounted for by the mechanical irritation and pressure upon the tissues produced by their rapid multiplication, and by actual trauma of the cell walls perforated by sharp projections of the bacterium. This compression produced disturbance of nutrition, obstruction of circulation, interference with the regular excretion of the effete residues of tissue metamorphosis. Added to this was the using up of the nutrient elements required for the building up and repair of the body. All such consequences of bacterial invasion appear too natural and self-evident not to be assented to without much contradiction. But how does this explain the manifold symptoms observed at the bedside of persons affected with infectious disease? If the causes admitted to exist were the only or even the most important ones, whence the great multiplicity of phenomena? Why not identical, or at least very similar, disturbances when the same organ is infected by the most different parasites? We might thus account for a difference in the intensity of the symptoms, but never for the great variety actually observed.

Let us consider the action of bacteria and living ferments on dead material. In a solution of glucose quite a number of various saccharomyces grow and multiply at the expense of the sugar, leaving behind carbonic acid and alcohol as the final products of their vital process. In the same sugar solution bacillus subtilis effects quite a different metamorphosis, and lactic and butyric acids are here the result of the fermentation. Glycerine by the same bacillus is changed to propylic alcohol. If the brewer permits the true beer yeast, saccharomyces cerevisiæ, in his malt infusion to be substituted by other closely related cryptococci (known as spurious yeast) then alcohol is still formed, but is accompanied by nauseous division products of the sugar, and the beer is spoilt. Wine, fermented with spurious yeast becomes stringy or acrid or otherwise deteriorated, as is ably shown in Pasteur's investigations. It would be easy to cite other well-known examples of similar character. We find here evidently that, when studying bacterial action, we must regard not only the material used up for their nutrition, but also the excrementitious residues they leave behind them.

The bacteria which excite malodorous putrefaction produce besides carbonic acid gas, sulphuretted hydrogen and gaseous hydrocarbons, also phenol, ortho- and para-cressol, indol, scatol, and, according to the different composition of the tissues invaded, a variety of ptomaines, muscarine, putrescine, cadaverine, gadinine, etc., as already stated.

Very similar to this is the action of pathogenous bacteria so far as at present investigated.

In 1885, Villiers isolated, according to Brieger's method, from bodies dead with *cholera*, a liquid ptomaine possessing the odor of hawthorne flowers, which in experimental animals produced violent tremors and strong disturbances of the heart's action, together with increased peristaltic action of the intestines.

He afterwards found a peculiar ptomaine in the victims of *pneumonia*, which bore close resemblance to that found in cases of *diphtheria*, but neither of those preparations was obtained in sufficient quantity to obtain its pure and crystallized salts for analysis.

Hoffa and others studied a ptomaine produced by the *bacillus anthracis*, but did not succeed in arriving at an unquestionable result.

The interesting researches of A. Poehl show that various bacteria, inoculated into sterilized gelatine, so changed this material that it gave undoubted evidence of the production of reducing ptomaines, whose formation could be observed while the life-process of the bacteria was going on. Among these were the bacillus of typhus, the streptococcus pyogenes aureus, Koch's comma bacillus (brought by Dr. Raptschewsky from Spain). All these strongly reduced the ferric ferricyanide added to the gelatine, while the Prior - Finkler bacillus of cholera nostras, in spite of repeated trials, yielded no such reducing product, and throughout showed itself much less greedy of oxygen than the bacillus of cholera Asiatica. The experiments, however, do not exclude the formation of a non-reducing ptomaine by the Prior-Finkler bacillus of cholera nostras (the vibro proteus). The comma bacillus also produced in these experiments a peculiar red pigment, soluble in amylic alcohol, whose composition is most probably that of a derivative of scatol (methyl-indol).

Professor Brieger, a very zealous worker in this field, speaks of this relation of the bacteria in the third volume of his *Untersuchungen ueber die Ptomaine.* The changeable picture presented by infectious diseases points out that this group of maladies, comprising the far preponderating majority of all cases of sickness, must seek its origin in very different causes. Quite a number of different pathogenic bacteria are already known, whose agency in the causation of a series of diseases is beyond all controversy.

He dwells on the necessity of discarding ambiguous terms, to which, by various succeeding writers, different shades of meaning have been affixed, and advocates the adoption of a clear, well-defined nomenclature so as to avoid future misunderstandings. He proposes the retention of the generic name of ptomaine for any basic product of bacterial action, while he gives the names of toxines to the poisonous ptomaines. The name leucomaines is reserved for the bases examined by Gautier and by Kossel and Salomon, which are formed from the albumens during the normal process of life.

To avoid confusion, Brieger first re-examined all the ptomaines produced by the action of the bacteria of putrefaction (B. termo and B. lineola), among which there are found several highly poisonous toxines. In addition to those already mentioned, he found in putrid horse-flesh (of which sometimes several hundred pounds were used at once), besides cadaverine and putrescine, a very poisonous amido-acid, $C_7H_{17}NO_{21}$, which produced in experimental animals symptoms resembling those by curare. This was accompanied by a still more poisonous, but more slowly acting base, the mydatoxine, $C_6H_{13}NO_2$; also the poisonous methyl - guanidine, $C_2H_7N_3$, evidently a derivative of kreatine.

Oscar Bocklisch, together with Brieger, examined putrefying fish and found the non-poisonous bases, cadaverine and neuridine; besides these, di- and tri-methylamine, putrescine and other putrefaction bases.

In the mytilus edulis, an oyster-like mollusk, whose use as food has several times occasioned extensive poisoning, Brieger found the highly poisonous mytilotoxine, $C_6H_{16}NO_2$ betaine and other bases, as the products of incipient putrefaction.

After a very thorough-going study of the products of the putrefactive process, Brieger investigated the ptomaines produced by the culture on sterilized substrata of pathogenic bacteria.

Staphylococous pyogenes aureus yielded no toxine, but only ammonium salts. Pure cultures of streptococcus yielded considerable quantities of tri-methyl-amine, which is not entirely free from poisonous properties.

The Koch-Eberth typhus bacillus produced a small quantity of an exceedingly poisonous ptomaine, having the formula $C_7H_{17}NO_2$ (isomeric, but not identical with the above mentioned putrefaction ptomaine). To this the name typhotoxine was given. It produced in experimental animals a lethargic condition and other symptoms resembling those of typhus in man.

From cultures of the tetanus virus Brieger obtained the base tetanine $C_{13}H_{30}N_2O_4$, besides much ammonia. The hydrochlorate and still more the free base, when injected into the lower animals, produced clonic and tonic spasms of great violence, and led rapidly to a fatal termination.

Of others who have investigated this interesting subject, I mention Doleris and Butte, who in the blood of eclamptic patients found a crystalline toxine.

Bouchardat studied the phenomena of poisoning produced by injected urine. Bocklisch confirms the absence of ptomaines in the pure cultures of the vibrio proteus, the Prior-Finkler bacillus of cholera nostras. Besides these, many others are giving their time and talents to this problem.

Brieger announces that he will continue his researches, and probably ere long we may hear of further important discoveries.

In the light of such revelations the endeavor of the physician will henceforth not be confined merely to the prevention of infectious diseases, or the destruction of the parasitic organisms, but also to the discovery of suitable antidotes to the poisonous compounds introduced into the system by their agency.—*Weekly Medical Review.*

